# Simultaneous Quantification of Multiple Representative Components in the Xian-Ling-Gu-Bao Capsule by Ultra-Performance Liquid Chromatography Coupled with Quadrupole Time-of-Flight Tandem Mass Spectrometry

**DOI:** 10.3390/molecules22060927

**Published:** 2017-06-02

**Authors:** Zhi-Hong Yao, Zi-Fei Qin, Hong Cheng, Xiao-Meng Wu, Yi Dai, Xin-Luan Wang, Ling Qin, Wen-Cai Ye, Xin-Sheng Yao

**Affiliations:** 1 College of Pharmacy, Jinan University, Guangzhou 510632, China; qzf1989@163.com (Z.-F.Q.); chenghong19880216@163.com (H.C.); xiaomengwu2011@163.com (X.-M.W.); chyewc@gmail.com (W.-C.Y.); tyaoxs@jnu.edu.cn (X.-S.Y.); 2 Guangdong Provincial Key Laboratory of Pharmacodynamic Constituents of TCM and New Drugs Research, College of Pharmacy, Jinan University, Guangzhou 510632, China; 3 G Integrated Chinese and Western Medicine Postdoctoral Research Station, Jinan University, Guangzhou 510632, China; 4 Musculoskeletal Research Laboratory, Department of Orthopedics and Traumatology, the Chinese University of Hong Kong, Satin, N.T. Hong Kong SAR, China; xl.wang@siat.ac.cn (X.-L.W.); qin@ort.cuhk.edu.hk (L.Q.)

**Keywords:** Xian-Ling-Gu-Bao capsule, representative components, quantification, quality control, UPLC/Q-TOF-MS

## Abstract

Xian-Ling-Gu-Bao capsule (XLGB), a famous traditional Chinese medicine prescription, is extensively used for the treatment of osteoporosis in China. However, few studies on the holistic quality control of XLGB have been reported. In this study, a reliable method using 18 representative components in XLGB was successfully established and applied to evaluate 34 batches of XLGB samples by ultra-performance liquid chromatography coupled with quadrupole time-of-flight mass spectrometry (UPLC/Q-TOF-MS). The choice of quantitative markers mostly followed four principles, i.e., absorbed components in plasma, bioactive compounds with in vitro anti-osteoporosis activity, those derived from multiple individual medicinal herbs in XLGB with multiple representative structure types, and quantitative chemical markers in the Chinese Pharmacopoeia. The results showed chemical consistency was good except for individual batches. Multivariate statistical analysis indicated that asperosaponin VI from Radix Dipsaci, epimedin C, magnoflorine, and icariin from Herba Epimedii as well as timosaponin BII from Rhizoma Anemarrhenae varied significantly in multiple samples, which hinted an assay for these four components should be completed during all of the manufacturing processes. Taken together, this study provided a feasible method for holistic quality control of XLGB by multiple chemical markers, which could play a vital role in guaranteeing the safety, effectiveness, and controllability of administering the capsules as a medication in clinics.

## 1. Introduction

Over the last few decades, traditional Chinese medicines (TCMs) have gained increasing globalization and use worldwide for human health care due to their long clinical practice in treating chronic and complex diseases [1,2]. As TCMs are growing in popularly, the quality control of TCMs has become more crucial than ever [3,4]. It is well known that TCMs or traditional Chinese medicine prescriptions (TCMPs) exert effects via a holistic mode of multiple-components and multiple-targets. The discovery of multiple bioactive components could be reasonably performed by in vivo metabolite profiling and in vitro efficacy-associated activity evaluation. Hence, the multiple bioactive components that contribute most to the efficacies of TCMs or TCMPs should be selected as representative constituents for developing the quality control method [5,6]. 

The Xian-Ling-Gu-Bao capsule (XLGB), the anti-osteoporosis TCMP listed in the 2017 edition of the China National Basic Drugs Catalogue [7], is widely used for the prevention and treatment of osteoporosis [8,9]. It consists of the following six commonly used TCMs: Herba Epimedii (70%), Radix Dipsaci (10%), Fructus Psoraleae (5%), Rhizoma Anemarrhenae (5%), Radix Salviae Miltiorrhizae (5%), and Radix Rehmanniae (5%) [10]. In addition, the XLGB has been proven to be safe and effective for the treatment of osteoporosis in postmenopausal women by the Evidence-Based Medicine test [11]. Meanwhile, pharmacological research showed that the XLGB could protect musculoskeletal tissues in old and ovariectomized rats as well as preventing bone loss in ovariectomized mice by inhibiting bone remodeling [12,13,14]. Moreover, no adverse effects were observed in rats after the oral administration of the XLGB at a dose of 1000 mg/kg, which is equivalent to 3.3 times the human dose based on the conversion of body surface area [15]. In view of its notable curative effects and widespread use, quality control of the XLGB deserves more attention. 

To date, numerous studies concentrating on quality control of the XLGB focused on only one or two individual medicinal herbs (mainly Herba Epimedii and Fructus Psoraleae) [16,17,18,19]. As the capsule consists of six individual medicinal herbs and various types of chemical constituents, the holistic feature of the XLGB should not be characterized by the content of only one or several representative bioactive ingredients from one or two individual medicinal herbs, which cannot be proven to be associated with its clinical efficacy [20]. Therefore, a holistic quality control of multiple representative components with bioactivities derived from multiple medicinal herbs in the XLGB is a necessity. 

In our previous study, sixty-one compounds were isolated and identified based on nuclear magnetic resonance (NMR) data from the bioactive fractions of the XLGB [21]. Furthermore, a total of 118 compounds were identified or tentatively characterized by LC-linear ion trap/orbitrap mass spectrometry [22]. To classify the effective components, an in vitro anti-osteoporosis evaluation [21,23] and in vivo metabolism in rats [24,25,26] were also investigated. These works provided a solid experimental foundation for the selection of representative chemical markers for the holistic quality evaluation of the XLGB. 

In this study, a total of 18 chemical markers were selected according to the following four principles: being absorbed in the plasma; possessing anti-osteoporosis activity in an in vitro test; feature representative chemical structures derived from different medicinal herbs in the capsule; and commercially available with the quantitative chemical markers for each medicinal herb in the composition recorded in the Chinese Pharmacopoeia [27]. Furthermore, an ultra-performance liquid chromatography coupled with quadrupole time-of-flight mass spectrometry (UPLC/Q-TOF-MS) method was developed and successfully applied to determine the contents of multiple representative chemical markers to evaluate the consistency of multiple XLGB samples. Moreover, chemometric analyses, such as principal component analysis (PCA), were utilized to define the main relevant variables that contributed most to differences evaluation. These results would provide a practical approach to evaluate the quality of multiple XLGB samples. During all of the manufacturing processes, the main relevant variables should be monitored. This would help to improve the consistency of multiple XLGB samples, which would play a vital role in their safe clinical administration as medication.

## 2. Results and Discussion

### 2.1. Selection of Quantitative Chemical Markers

For quantitative chemical markers, four main principles were followed. First, they were components absorbed in vivo. Generally, the components absorbed in vivo were considered as potential directly effective materials for therapeutic effects. As previously reported, a total of 57 prototype components were detected in rat plasma after the oral administration of XLGB extracts [24].

Second, the quantitative chemical markers should also exhibit the same or similar activity as indicative of the TCMP. On the premise that abundant amounts of chemical components in TCM and TCMP are available, and that several components which possess activity in vitro, exhibit good absorption properties in vivo, and maintain a certain concentration in target organs for a finite period of time, then further activity evaluation in vivo could be performed. On the basis of our previous study or other related studies, several markers showed obvious anti-osteoporosis bioactivity in vitro [21,23]. Other compounds were reported with oestrogenic activities and osteoblast proliferation and differentiation-stimulating activities [28,29,30,31]. 

Third, in consideration of the quality control of TCMPs, the quantitative chemical markers should be derived from different individual medicinal herbs in the TCMP. The XLGB includes six common TCMs. Meanwhile, there were numerous components with multiple representative structure types, such as prenylated flavonoids and alkaloids from Herba Epimedii, saponins and iridoids from Radix Dipsaci, prenylated flavonoids and coumarins from Fructus Psoraleae, saponins from Rhizoma Anemarrhenae, and diterpenoid quinone from Radix Salviae Miltiorrhizae. To better evaluate the overall quality of the XLGB, the quantitative marker of the XLGB should be derived from as many individual medicinal herbs with multiple characteristic chemical structures as possible. 

Another important point is that the quantitative markers of different individual medicinal herbs in the XLGB according to the Pharmacopoeia of the Peoples’ Republic of China (2015 edition) [27] should be taken into account to ensure the improvement of the practicality of the method. Hence, except for salvianolic acid B from Radix Salviae Miltiorrhizae and catalpol as well as acteoside from Radix Rehmanniae, six other components (icariin, asperosaponin VI, psoralen, isopsoralen, timosaponin BII, and tanshinone IIA) from five of the six individual medicinal herbs were selected as quantitative chemical markers. 

**Figure 1:**
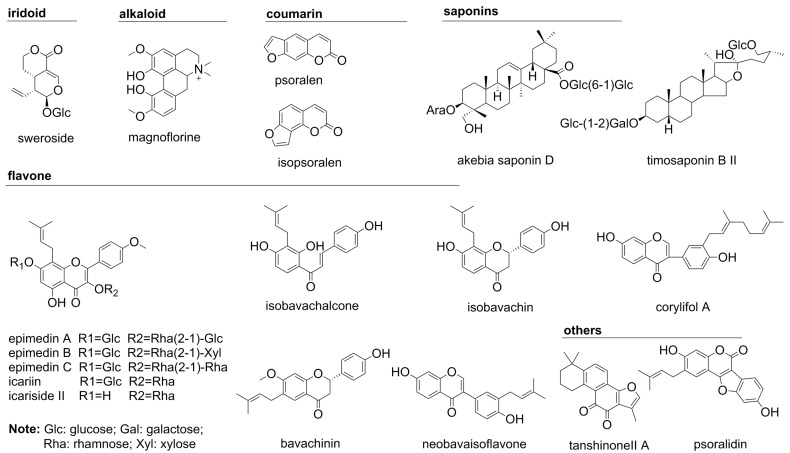
Structures of eighteen representative chemical markers in the Xian-Ling-Gu-Bao capsule (XLGB).

The detailed selection principles are shown in Table 1. As a result, a total of 18 compounds (magnoflorine, epimedin A, epimedin B, epimedin C, icariin, icariside II, psoralen, isopsoralen, isobavachin, neobavaisoflavone, psoralidin, isobavachalcone, bavachinin, corylifol A, sweroside, asperosaponin VI, tanshinone IIA and timosaponin BII) including one alkaloid and five prenylated flavonoids from Herba Epimedii, one iridoid and one saponin from Radix Dipsaci, one saponin from Rhizoma Anemarrhenae, five prenylated flavonoids and three coumarins from Fructus Psoraleae and one diterpenoid quinone from Radix Salviae Miltiorrhizae were considered to be quantitative chemical markers to represent the holistic quality of the XLGB. Due to the extremely complex composition of the XLGB and diverse structures of natural products, it is feasible that some chemical components from one individual medicinal herb (such as Radix Rehmanniae in the XLGB) failed to be screened out by this approach. Despite such a limitation, it would not restrict the application of this strategy because the quantitative markers were derived from most of the medicinal herbs of the XLGB. Their chemical structures are displayed in Figure 1 and their extracted ion chromatograms (EICs) shown in Figure S1.

### 2.2. Method Validation

The validation of the method included selectivity, limit of detection (LOD), and limit of quantification (LOQ), linearity, precision, repeatability, recovery, and stability tests. All measurements for method validation followed the guidelines of the Pharmacopoeia of the Peoples’ Republic of China (2015 edition) [27].

#### 2.2.1. LODs, LOQs, and Linearity

The limits of detection (LODs) and limits of quantification (LOQs) were measured with signal to noise ratios (*S*/*N*) of 3 and 10 as criteria, respectively. The linear calibration curves, plotted with a series of seven concentrations of reference standard solutions, were constructed from the peak area of each bioactive component (y) versus the concentration of each analyte (x).

As a result, all the calibration curves showed good linear correlation, with the correlation coefficients (*r*^2^) no less than 0.9990 within the test ranges. As shown in Table 2, the LODs were 0.42–40.17 ng/mL, while the LOQs were 1.19–114.33 ng/mL.

#### 2.2.2. Intra- and Inter-Day Precision

Intra-day and inter-day variations were determined to assess the precision of the method. The intra-day variation was examined six times in one day, and the inter-day variation was analyzed on three successive days. To check the repeatability, six replicates of the same XLGB sample (No. 110319) were determined. The results showed that the relative standard deviations (RSDs) of the intra- and inter-day precisions were below 3.9% and 3.7%, respectively. The detailed information is displayed in Table 3.

#### 2.2.3. Recovery and Stability

To examine the accuracy of the assay, the determination of the recovery was carried out by the standard addition method at three concentration levels. Standard mixtures of three concentration levels (80%, 100%, and 120%) were added into the sample solution (No. 110319), with three replicates for each level. Recoveries were calculated by the following formula: recovery (%) = (observed amount – original amount)/spiked amount × 100. Variations were expressed as the percentage RSD. The mean recoveries varied from 96.3% to 104.5% with RSDs within 4.9% (shown in Table 4).

In addition, the same XLGB sample (No. 110319) was analyzed at 0, 12, 24, 36, 48, and 72 h after extraction to examine the stability of the sample. The stability test showed that the sample solution was stable for 72 h with an RSD less than 3.0% (Table 4).

### 2.3. Sample Determination

**Figure 2:**
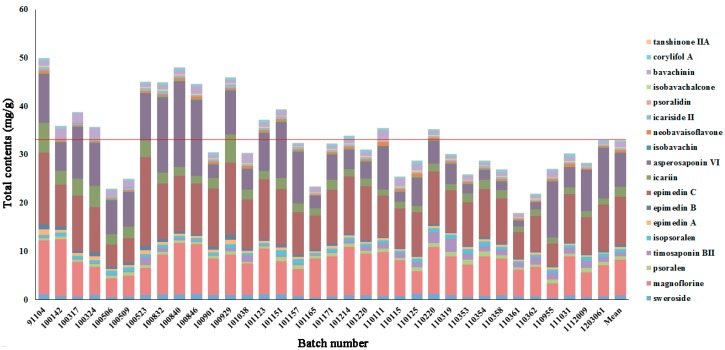
Total contents (mg/g) of eighteen representative chemical markers in 34 batches of XLGB samples.

This newly developed and validated method was applied to 34 batches of XLGB samples and the quantification results are summarized in Table S1. A graphical comparison of the contents of 18 quantitative chemical markers in 34 XLGB samples is exhibited in Figure 2. Not surprisingly, it was discovered that all of the samples contained a high level of magnoflorine, epimedin C, asperosaponin VI, and icariin. For the high level of icariin, it was mandatory for the manufacturers to meet the standards by State Drug Administration. The standard stipulates that the content of icariin shall not be less than 1.5 mg per capsule. The pharmacological actions of these four components are directly associated with those of the whole TCMP (shown in Table 1), suggesting that if there were additional analytes they should be considered for inclusion in Chinese pharmacopeia standards [27], magnoflorine and epimedin C would be a preference. 

Furthermore, assay results showed that the contents of some components in each sample ranged significantly. Obviously, the total contents of several XLGB samples were higher than others, such as batch number 91104, 100523, 100832, 100840, 100846, and 100929, while the total contents of some XLGB samples (batch number 100506, 100509, 101165, 110115, 110361, and 110362) were significantly lower. The marked different consistency of multiple XLGB samples exhibited in a random pattern. As clearly shown in Figure 2, the contents of several components varied significantly, such as asperosaponin VI, epimedin C, and magnoflorine. The content ranges of asperosaponin VI, epimedin C, magnoflorine, and icariin in 34 batches of XLGB capsule were 1.242–17.549, 4.584–15.012, 2.930–11.729, and 1.134–6.110, respectively. The multiples of asperosaponin VI, epimedin C, magnoflorine, icariin, and timosaponin B II were 14.2, 3.3, 4.1, and 5.4, respectively. To better determine the markers that contributed most to the fluctuation among multiple XLGB samples, a relevant multivariate statistical analysis was conducted.

### 2.4. Multivariate Statistical Analysis

Chemometric analysis, using techniques such as principal component analysis (PCA), has become an effective approach in the quality evaluation of TCMs and TCMPs [32,33]. From the PCA score plot, significant separation among different samples was observed, and the loadings plot could pick out the potential discriminatory components from differences evaluation as well. 

**Figure 3:**
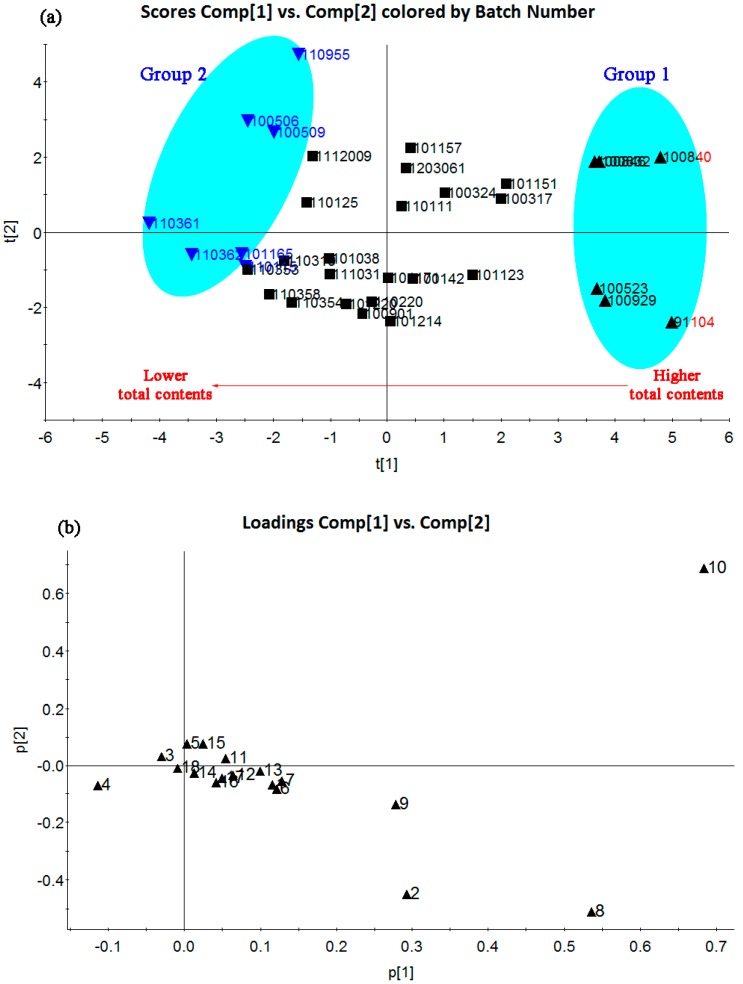
Score scatter plot (**a**) of 34 XLGB samples and loading plot (**b**) of eighteen representative chemical markers in the XLGB samples. (1: sweroside; 2: magnoflorine; 3: psoralen; 4: timosaponin BII; 5: isopsoralen; 6: epimedin A; 7: epimedin B; 8: epimedin C; 9: icariin; 10: asperosaponin VI; 11: isobavachin; 12: neobavaisoflavone; 13: icariside II; 14: psoralidin; 15: isobavachalcone; 16: bavachinin; 17: corylifol A; 18: tanshinone IIA)

In this study, PCA was performed with data preprocessing of scaling by the software EZinfo software of the Masslynx V4.1 workstation (Waters, Milford, MA, USA) based on the contents of 18 chemical markers in 34 batches of XLGB samples. A two-component PCA model was obtained which cumulatively accounted for 69.0% of the variation; the total variance explained for the first principal component is 43.9% and that for the second principal component is 25.1%. Through a visual analysis of Figure 3a, separations among three sets of samples were observed in the PCA plot. Group 1, including samples 91104, 100523, 100832, 100840, 100846, and 100929, was clearly different from the other two groups. Group 2 (samples 110361, 110362, 100506, 100509, 110955, 101165, and 110115) and the other XLGB samples were slightly overlapping. The tendency of total contents of 18 analytes was degressive from the samples present on the right side of Figure 3a to the samples on the left side. 

Furthermore, the loadings plot (Figure 3b) could clearly give the highest contributing components that contribute to the differences evaluation. The variables with larger covariance and correlation values were most likely considered discriminatory components and more relevant to sample classification. Obviously, epimedin C, magnoflorine and icariin from Herba Epimedii, asperosaponin VI from Radix Dipsaci as well as Timosaponin B II from Rhizoma Anemarrhenae were the five most important compounds with good confidence intervals to distinguish differences among 34 batches of XLGB samples. There were a variety of reasons for sample differences, such as the different harvest time, geographical environment, climates, herb processing methods, etc. Thus, the contents of these main relevant variables should be focused on during the manufacturing process. 

In addition, only icariin from Herba Epimedii in XLGB samples has been considered so far as the quantitative marker for the quality evaluation in the standards by State Drug Administration. Actually, if there were only one compound considered for quality control, the holistic nature of TCMs or TCMP would not be emphasized [34,35]. By referring to the standard of the Chinese pharmacopoeia [27], it transpired that it was possible to determine whether six of the TCMs in TCMP were qualified or not, through quantification of their particular constituents. Icariin, epimedin C, and magnoflorine from Herba Epimedii, asperosaponin VI from Radix Dipsaci, timosaponin BII from Rhizoma Anemarrhenae, tanshinone IIA from Radix Salviae Miltiorrhizae, pesoralen and isoralen from Fructus Psoraleae should all be defined as quantitative markers to evaluate the quality of XLGB samples. 

This study illustrates that quality control of XLGB is urgently needed of multiple components quantification. This would benefit the promotion of the standards by State Drug Administration. Theoretically, if at least one component of each TCM in ae XLGB capsule could be monitored and quantified in the manner accomplished in this study, then the TCM manufacturers would have to ensure the quality of every TCM, and such a loophole would be significantly eliminated, and patient care improved. 

## 3. Materials and Methods 

### 3.1. Materials and Reagents

Thirty-four batches of XLGB samples (batch number: 091104, 100142, 100317, 100324, 100506, 100509, 100523, 100832, 100840, 100846, 100901, 100929, 101038, 101123, 101151, 101157, 101165, 101171, 101214, 101220, 110111, 110115, 110125, 110220, 110319, 110353, 110354, 110358, 110361, 110362, 110955, 111031, 1112009, and 1203061) that were manufactured by Guizhou Tongjitang Pharmaceutical Co., Ltd. (Guiyang, China) were collected from drugstores in Guangzhou of China. Epimedin A, epimedin B, asperosaponin VI, isobavachin, neobavaisoflavone, icariside II, bavachinin, isobavachalcone, sweroside, and corylifol A were purchased from Shanghai Winherb Medical Technology Co., Ltd. (Shanghai, China). Psoralen, timosaponin BII, isopsoralen, epimedin C, icariin, psoralidin, and tanshinone II A were obtained from Guangzhou PI&PI Inc. (Guangzhou, China). Magnoflorine was obtained from Sigma-Aldrich (St. Louis, USA). Their purities were all determined to be over 98% by HPLC. 

LC-MS grade methanol, acetonitrile and water were all purchased from Fisher Scientific (Pittsburgh, PA, USA). LC-MS grade formic acid was purchased from Sigma-Aldrich (St. Louis, MO, USA). Other reagents were of analytical grade.

### 3.2. Sample Preparation

An adequate amount of each reference standard was dissolved in 60% methanol to prepare the standard solution. Subsequently, these reference standard solutions were mixed together to achieve a reasonable concentration. Before use, all of the reference standard solutions were stored at 4 °C. 

The powder of the XLGB was extracted by ultrasonic extraction with 60% aqueous methanol, and then, a final concentration of 2.5 mg/mL was achieved. After centrifugation at 13,800× *g* for 1 min, the supernatants were filtered through a 0.22 µm membrane filter. Then, 2 µL samples were injected into the UPLC/Q-TOF-MS system. All of the solutions were stored at 4 °C before analysis. 

### 3.3. UPLC Conditions

The analysis was performed on an ACQUITY^TM^ UPLC I-Class system (Waters Corporation, Manchester, UK) equipped with a binary solvent system, an automatic sample manager, and a photo-diode array (PDA) detector. The separation was carried out on an ACQUITY UPLC^TM^ BEH C18 column (3.0 × 150 mm, 1.7 µm, Waters Corporation, Manchester, UK) at a temperature of 35 °C. The mobile phase consisted of mobile phase A (0.1% formic acid in water, *v*/*v*) and mobile phase B (0.1% formic acid in acetonitrile, *v*/*v*) at a flow of 0.6 mL/min. The non-linear gradient elution program was as follows: 2% B from 0 to 0.11 min, 2–15% B from 0.11 to 4.2 min, 15–20% B from 4.2 to 4.5 min, 20–25% B from 4.5 to 9 min, 25–35% B from 9 to 15 min, 35–75% B from 15 to 16 min, 70–100% B from 16 to 16.5 min, and 100% B from 16.5 to 17.5 min. The injection volume was 2 µL. 

### 3.4. Q-TOF/MS Conditions

The UPLC system was coupled to a hybrid quadrupole orthogonal time-of-flight (Q-TOF) mass spectrometer (SYNAPT^TM^ G2 HDMS, Waters, Manchester, UK) equipped with electrospray ionization (ESI). The ionization was acquired in the positive mode. The operating parameters were as follows: capillary voltage of 3 kV (ESI+ ); sample cone voltage of 35 V; extraction cone voltage of 4 V, source temperature of 100 °C, desolvation temperature of 300 °C, cone gas flow of 50 L/h; and desolation gas flow of 800 L/h. The retention time, precursor ion, daughter ion, and collision energy under selective ion monitoring (SIM) and multiple reaction monitoring (MRM) modes are all shown in Table 5. All experimental data were collected in the centroid mode and processed using Masslynx^TM^ 4.1 software with a Quanlynx^TM^ program. 

## 4. Conclusions

In this study, a strategy of choosing quantitative markers regarding the holistic quality control of a TCMP has been successfully developed and exemplified for the simultaneous quantification of 18 representative components derived from most of the medicinal herbs of the XLGB by UPLC-Q-TOF/MS. Meanwhile, the combination of major representative chemical components from multiple individual medicinal herbs and in vivo absorbed bioactive components will be inevitably increasingly popular for the selection of quantitative markers when we hope to establish an overall quality control-oriented assay for their contents in TCMPs. In addition, this method could distinguish the consistency of multiple XLGB samples based on the quantified measurement of 18 analytes, thereby ensuring the quality of XLGB samples. Asperosaponin VI from Radix Dipsaci, icariin, epimedin C and magnoflorine from Herba Epimedii, timosaponin BII from Rhizoma Anemarrhenae, were all recommended as quantitative markers for quality evaluation of XLGB samples. These findings demonstrated that it was imperative to apply the simultaneous quantification of multiple components to the quality control of XLGB samples, so that the corresponding standards could be promoted and the quality could be ensured. This study is expected to be universally applicable to the holistic quality control of other TCMs and TCMPs.

## Figures and Tables

**Table 1:** Eighteen representative chemical markers in the Xian-Ling-Gu-Bao capsule (XLGB) selected for simultaneous quantitative determination

No.	Compounds	Absorbed in Vivo	Activity in Vitro	CH.P Markers	Type	Res.	Ref.
1	sweroside	√	√ ^a^	/	iridoid	R.D.	[21,23]
2	magnoflorine	√	√ ^a^	/	alkaloid	H.E.	[20,22]
3	psoralen	√	√ ^a^	√	coumarin	F.P.	[20,22]
4	timosaponin BII	√	/	√	saponin	R.A.	
5	isopsoralen	√	/ ^a^	√	coumarin	F.P.	[20,22]
6	epimedin A	√	√ ^a^	/	prenylated flavonoid	H.E.	[20,22]
7	epimedin B	√	√ ^a^	/	prenylated flavonoid	H.E.	[20,22]
8	epimedin C	√	√ ^a^	/	prenylated flavonoid	H.E.	[20,22]
9	icariin	√	√ ^a^	√	prenylated flavonoid	H.E.	[20,22]
10	asperosaponin VI	√	√ ^a^	√	saponin	R.D.	[20,22]
11	isobavachin	√	√ ^a^	/	prenylated flavonoid	F.P.	[20,22]
12	neobavaisoflavone	√	√ ^a^	/	prenylated flavonoid	F.P.	[20,22]
13	icariside II	√	√ ^a^	/	prenylated flavonoid	H.E.	[20,22]
14	psoralidin	√	√ ^b^	/	coumarin	F.P.	[28]
15	isobavachalcone	√	√ ^b^	/	prenylated flavonoid	F.P.	[29]
16	bavachinin	√	√ ^b^	/	prenylated flavonoid	F.P.	[30]
17	corylifol A	√	√ ^b^	/	prenylated flavonoid	F.P.	[31]
18	tanshinone IIA	√	/	√	phenanthraquinone	R.S.	

Notes: ^a^ indicated that the compounds were tested with anti-osteoporosis activity in vitro by our laboratory [21,23]. ^b^ indicated that the compounds were reported with anti-osteoporosis activity in the literature [28,29,30,31]. CH.P: Chinese Pharmacopoeia (2015 edition); R.D.: Radix Dipsaci; H.E.: Herba Epimedii; F.P.: Fructus Psoraleae; R.A.: Rhizoma Anemarrhenae; R.S.: Radix Salviae Miltiorrhizae. Res. means the resource of Chinese herbal medicine. Ref. means the cited references.

**Table 2:** Linear ranges, calibration curves, correlation factors, limits of detection (LODs), and limits of quantification (LOQs) of the eighteen representative chemical markers in the XLGB.

No.	Compounds	Linear Range (μg/mL)	Calibration Curves	*r*^2^	LODs (ng/mL)	LOQs (ng/mL)
1	sweroside	0.34–6.72	y = 29.93x + 10.53	0.9995	18.40	46.00
2	magnoflorine	1.54–30.74	y = 547.76x + 577.25	0.9990	4.59	10.20
3	psoralen	0.20–4.06	y = 609.97x + 7.73	0.9993	7.64	22.92
4	timosaponin BII	0.42–8.44	y = 180.05x + 1.04	0.9991	40.17	114.33
5	isopsoralen	0.26–5.10	y = 483.61x + 41.92	0.9998	8.85	17.70
6	epimedin A	0.24–4.90	y = 220.86x + 21.40	0.9995	2.90	8.68
7	epimedin B	0.22–4.39	y = 275.24x + 38.12	0.9992	15.45	30.90
8	epimedin C	4.47–89.36	y = 189.36x + 999.16	0.9991	5.40	10.80
9	icariin	1.46–29.11	y = 498.73x − 239.20	0.9991	21.80	52.50
10	asperosaponin VI	2.26–45.17	y = 12.72x + 27.954	0.9993	40.00	100.00
11	isobavachin	0.05–1.06	y = 508.91x + 1.72	0.9994	1.56	5.20
12	neobavaisoflavone	0.20–4.07	y = 845.42x + 64.26	0.9997	1.38	5.30
13	icariside II	0.15–2.97	y = 1191.92x + 124.27	0.9993	5.50	16.50
14	psoralidin	0.02–0.47	y = 1093.91x + 49.45	0.9991	0.98	1.96
15	isobavachalcone	0.11–2.28	y = 1145.10x + 110.41	0.9990	23.60	59.00
16	bavachinin	0.30–5.91	y = 76.00x + 6.84	0.9998	5.08	10.15
17	corylifol A	0.15–3.01	y = 1111.52x − 8.71	0.9991	0.80	2.28
18	tanshinone IIA	0.01–0.19	y = 9055.01x + 117.51	0.9994	0.42	1.19

**Table 3:** The relative standard deviation (RSD) values of intra- and inter-day precisions of the 18 chemical markers in XLGB.

No.	Compounds	Conc. (μg/mL)	Intra-day (*n* = 6)		Inter-day (*n* = 3)	
			Mean ± SD (μg/mL)	RSD (%)	Mean ± SD (μg/mL)	RSD (%)
1	sweroside	0.34	0.35 ± 0.01	1.5	0.32 ± 0.01	1.7
		1.34	1.38 ± 0.03	2.3	1.28 ± 0.04	3.1
		4.03	4.23 ± 0.10	2.4	3.96 ± 0.05	1.2
2	magnoflorine	1.54	1.56 ± 0.04	2.3	1.46 ± 0.03	2.3
		6.15	6.26 ± 0.09	1.5	6.06 ± 0.03	0.5
		18.44	18.81 ± 0.15	0.8	17.62 ± 0.60	3.4
3	psoralen	0.20	0.22 ± 0.01	3.6	0.19 ± 0.00	0.9
		0.81	0.87 ± 0.01	0.8	0.81 ± 0.02	2.4
		2.44	2.56 ± 0.07	2.6	2.23 ± 0.06	2.6
4	timosaponin BII	0.42	0.45 ± 0.01	2.4	0.42 ± 0.01	2.1
		1.69	1.78 ± 0.07	3.9	1.72 ± 0.02	1.2
		5.06	5.46 ± 0.10	1.8	5.04 ± 0.07	1.3
5	isopsoralen	0.26	0.28 ± 0.01	1.8	0.25 ± 0.00	1.5
		1.02	1.12 ± 0.03	2.6	0.99 ± 0.03	2.6
		3.06	3.56 ± 0.10	2.6	3.26 ± 0.02	0.7
6	epimedin A	0.24	0.25 ± 0.01	3.1	0.21 ± 0.00	1.8
		0.98	0.99 ± 0.02	2.2	0.91 ± 0.02	1.9
		2.94	3.02 ± 0.06	2.0	2.91 ± 0.07	2.5
7	epimedin B	0.22	0.26 ± 0.00	1.5	0.23 ± 0.01	2.9
		0.88	0.92 ± 0.02	1.9	0.86 ± 0.01	0.8
		2.63	2.84 ± 0.09	3.1	2.64 ± 0.05	1.9
8	epimedin C	4.47	4.57 ± 0.11	2.5	4.27 ± 0.07	1.7
		17.87	18.57 ± 0.61	3.3	18.07 ± 0.20	1.1
		53.62	55.72 ± 0.84	1.5	53.23 ± 0.28	2.4
9	icariin	1.46	1.56 ± 0.04	2.7	1.46 ± 0.03	2.3
		5.82	5.94 ± 0.12	2.0	5.12 ± 0.03	2.5
		17.45	18.17 ± 0.50	2.7	17.86 ± 0.29	1.6
10	asperosaponin VI	2.26	2.35 ± 0.04	1.6	2.15 ± 0.04	1.8
		9.03	9.54 ± 0.20	2.1	9.06 ± 0.17	1.9
		27.10	28.21 ± 0.25	0.9	27.92 ± 0.73	2.6
11	isobavachin	0.05	0.06 ± 0.00	3.4	0.05 ± 0.00	1.7
		0.21	0.23 ± 0.00	1.7	0.21 ± 0.01	2.7
		0.64	0.67 ± 0.02	2.4	0.58 ± 0.01	2.3
12	neobavaisoflavone	0.20	0.25 ± 0.00	1.9	0.21 ± 0.10	2.5
		0.81	0.85 ± 0.02	2.7	0.80 ± 0.03	3.4
		2.44	2.54 ± 0.02	0.7	2.44 ± 0.01	0.5
13	icariside II	0.15	0.17 ± 0.00	1.5	0.16 ± 0.00	0.5
		0.60	0.62 ± 0.01	1.9	0.58 ± 0.00	1.2
		1.78	1.85 ± 0.05	2.8	1.73 ± 0.03	1.8
14	psoralidin	0.02	0.02 ± 0.00	2.1	0.02 ± 0.00	2.6
		0.09	0.10 ± 0.00	3.4	0.09 ± 0.00	0.7
		0.28	0.30 ± 0.01	2.0	0.20 ± 0.00	1.1
15	isobavachalcone	0.11	0.13 ± 0.00	2.0	0.12 ± 0.00	1.8
		0.46	0.48 ± 0.11	2.3	0.42 ± 0.01	2.6
		1.37	1.45 ± 0.04	3.1	1.25 ± 0.04	3.2
16	bavachinin	0.30	0.33 ± 0.01	2.5	0.31 ± 0.10	2.9
		1.18	1.29 ± 0.02	1.9	1.04 ± 0.01	1.3
		3.55	3.68 ± 0.07	1.8	3.28 ± 0.04	1.3
17	corylifol A	0.15	0.18 ± 0.01	1.9	0.17 ± 0.00	1.5
		0.60	0.62 ± 0.01	2.2	0.57 ± 0.02	3.7
		1.81	1.95 ± 0.07	3.6	1.84 ± 0.05	2.6
18	tanshinone IIA	0.01	0.01 ± 0.00	2.7	0.01 ± 0.00	3.2
		0.04	0.04 ± 0.00	1.6	0.03 ± 0.00	0.5
		0.11	0.14 ± 0.00	1.8	0.13 ± 0.00	1.2

Note: Conc.: concentration.

**Table 4:** The RSD values of recovery and stability of the 18 chemical markers in XLGB.

No.	Compounds	Recovery (%) (*n* = 3)						Stability (*n* = 6)	
		Low		Medium		High		Mean ± SD (mg/g)	RSD (%)
		Mean	RSD	Mean	RSD	Mean	RSD		
1	sweroside	98.2	0.6	102.7	1.8	102.7	2.2	0.96 ± 0.02	2.1
2	magnoflorine	100.9	4.3	104.3	0.9	99.5	4.6	8.53 ± 0.04	0.5
3	psoralen	96.3	1.7	101.6	2.4	103.3	0.3	0.97 ± 0.03	3.0
4	timosaponin BII	98.3	4.6	100.3	1.8	100.5	3.4	2.86 ± 0.06	2.0
5	isopsoralen	98.6	1.6	104.0	1.3	103.5	0.2	0.80 ± 0.02	2.8
6	epimedin A	101.9	2.8	103.0	1.1	100.7	2.4	0.36 ± 0.01	2.4
7	epimedin B	103.0	1.7	100.5	1.2	100.8	2.4	0.28 ± 0.01	2.4
8	epimedin C	104.5	0.6	102.8	0.8	101.9	2.7	9.26 ± 0.16	1.7
9	icariin	96.6	1.0	101.6	1.8	100.4	3.7	1.57 ± 0.05	3.0
10	asperosaponin VI	100.3	3.2	99.0	0.9	99.7	3.5	4.32 ± 0.13	3.0
11	isobavachin	103.4	1.4	98.4	3.3	103.8	0.4	0.14 ± 0.00	2.6
12	neobavaisoflavone	102.4	0.6	98.6	1.9	103.2	1.5	0.43 ± 0.01	2.4
13	icariside II	98.1	3.1	102.5	1.4	102.3	2.3	0.24 ± 0.01	2.4
14	psoralidin	99.8	1.5	99.7	4.9	101.5	3.5	0.11 ± 0.00	0.8
15	isobavachalcone	100.1	2.2	99.2	2.1	101.3	4.2	0.25 ± 0.00	2.9
16	bavachinin	97.3	2.7	99.7	3.1	101.4	2.9	0.63 ± 0.02	1.1
17	corylifol A	96.8	2.1	96.8	1.8	102.3	3.7	0.32 ± 0.01	2.8
18	tanshinone IIA	99.8	2.8	101.7	4.1	101.8	0.6	0.04 ± 0.00	2.0

**Table 5:** Selective ion monitoring (SIM) and multiple reaction monitoring (MRM) transitions and optimized mass spectrometry parameters of 18 chemical markers in the ultra-performance liquid chromatography coupled with quadrupole time-of-flight mass spectrometry (UPLC/Q-TOF-MS) analysis.

No.	Compounds	*t*_R_ (min)	Precursor Ion (*m*/*z*)	Daughter Ion (*m*/*z*)	Collision Energy (eV)
1	sweroside	3.12	197.0824	127.0390	20
2	magnoflorine	3.24	342.1704	/	/
3	psoralen	5.83	187.0395	/	/
4	timosaponin BII	5.95	903.4953	/	/
5	isopsoralen	6.10	187.0395	/	/
6	epimedin A	6.44	839.2974	369.1338	30
7	epimedin B	6.57	809.2868	369.1338	30
8	epimedin C	6.78	823.3025	369.1338	35
9	icariin	6.89	677.2445	369.1338	35
10	asperosaponin VI	9.86	929.5110	437.3420	20
11	isobavachin	12.54	325.1440	149.0238	20
12	neobavaisoflavone	13.89	323.1283	267.0660	20
13	icariside II	15.02	369.1338	313.0710	25
14	psoralidin	15.83	337.1076	281.0468	20
15	isobavachalcone	16.08	325.1440	149.0246	20
16	bavachinin	16.15	339.1596	219.1026	25
17	corylifol A	16.23	391.1909	267.0660	20
18	tanshinone IIA	16.44	317.1154	/	/

## Supplementary Materials

The following are available online. Figure S1: Extracted ion chromatograms (EICs) of 18 chemical markers (a: Control solvent; b: Reference standards of 18 chemical markers; c: XLGB; 1: sweroside; 2: magnoflorine; 3: psoralen; 4: timosaponin BII; 5: isopsoralen; 6: epimedin A; 7: epimedin B; 8: epimedin C; 9: icariin; 10: asperosaponin VI; 11: isobavachin; 12: neobavaisoflavone; 13: icariside II; 14: psoralidin; 15: isobavachalcone; 16: bavachinin; 17: corylifol A; 18: tanshinone IIA), Table S1: Contents (mg/g) of 18 chemical markers in 34 batches of XLGB samples.
