# Supplementary material for: Simultaneous Quantification of Multiple Representative Components in the Xian-Ling-Gu-Bao Capsule by Ultra-Performance Liquid Chromatography Coupled with Quadrupole Time-of-Flight Tandem Mass Spectrometry
Source: Molecules. 2017 Jun 2;22(6):927. doi: 10.3390/molecules22060927 (PMC6152775; doi:10.3390/molecules22060927)
Supplement: Supplementary file 1 [file molecules-22-00927-s001.pdf]

Supporting Information： Simultaneous quantification of multiple representative components in the Xian-Ling-Gu-Bao capsule by ultra-performance liquid chromatography coupled with quadrupole time-of-flight tandem mass spectrometry

Legends

Table S1 Contents (mg/g) of 18 chemical markers in 34 batches of XLGB samples

Figure S1 Extracted ion chromatograms (EICs) of 18 chemical markers

(a: Control solvent; b: Reference standards of 18 chemical markers; c: XLGB; 1: sweroside; 2: magnoflorine; 3: psoralen; 4: timosaponin BII; 5: isopsoralen; 6: epimedin A; 7: epimedin B; 8: epimedin C; 9: icariin; 10: asperosaponin VI; 11: isobavachin; 12: neobavaisoflavone; 13: icariside II; 14: psoralidin; 15: isobavachalcone; 16: bavachinin; 17: corylifol A; 18: tanshinone IIA)

Table S1 Contents (mg/g) of 18 chemical markers in 34 batches of XLGB samples

Batches	1	2	3	4	5	6	7	8	9	10	11	12	13	14	15	16	17	18
**091104**	1.098	11.173	0.463	0.211	0.508	1.150	1.017	14.801	6.11	10.051	0.197	0.629	0.610	0.123	0.332	0.984	0.475	0.017
**100142**	0.747	11.729	0.572	0.217	0.621	0.630	0.637	8.618	2.856	5.844	0.130	0.383	0.348	0.056	0.048	2.062	0.376	0.023
**100317**	0.701	7.096	0.565	0.187	0.579	0.522	0.515	11.399	3.529	10.57	0.095	0.331	0.328	0.050	0.052	1.977	0.306	0.014
**100324**	1.027	5.830	0.534	0.219	0.592	0.835	0.870	9.277	4.437	8.754	0.122	0.340	0.394	0.057	0.065	2.039	0.330	0.017
**100506**	0.606	3.812	0.583	0.227	0.820	0.356	0.451	4.584	2.057	6.935	0.190	0.317	0.335	0.054	0.052	1.213	0.32	0.012
**100509**	0.668	4.374	0.674	0.175	0.873	0.451	0.509	4.994	2.402	7.049	0.192	0.344	0.354	0.050	0.059	1.463	0.331	0.005
**100523**	0.970	5.647	0.551	1.681	0.612	0.823	0.854	18.411	3.355	9.721	0.135	0.469	0.401	0.100	0.246	0.663	0.379	0.015
**100832**	1.131	8.211	0.606	0.799	0.663	0.538	0.572	11.482	2.354	15.296	0.251	0.659	0.470	0.120	0.332	0.971	0.504	0.019
**100840**	1.118	10.64	0.539	0.288	0.602	0.517	0.443	11.436	1.845	17.549	0.273	0.708	0.309	0.117	0.277	0.833	0.530	0.026
**100846**	1.209	10.258	0.488	0.205	0.576	0.437	0.346	10.543	1.610	15.469	0.265	0.618	0.279	0.094	0.217	1.648	0.305	0.022
**100901**	1.009	7.546	0.498	0.201	0.536	0.560	0.530	12.092	2.188	2.724	0.167	0.582	0.334	0.111	0.257	0.669	0.461	0.026
**100929**	1.040	8.334	0.694	0.671	0.754	0.928	0.979	15.012	5.770	8.928	0.165	0.497	0.732	0.094	0.244	0.677	0.359	0.021
**101038**	1.030	6.454	0.410	1.300	0.426	0.505	0.419	10.201	2.043	4.169	0.172	0.548	0.245	0.087	0.196	1.825	0.277	0.011
**101123**	1.160	9.413	0.465	0.243	0.464	0.448	0.383	12.397	1.644	7.655	0.207	0.643	0.271	0.100	0.307	0.909	0.48	0.01
**101151**	1.130	6.819	0.890	0.365	1.098	0.554	0.551	11.452	2.374	11.404	0.228	0.480	0.487	0.091	0.129	1.035	0.245	0.011
**101157**	0.773	5.639	0.789	0.273	1.004	0.404	0.396	8.845	1.795	10.553	0.232	0.443	0.378	0.087	0.127	0.388	0.277	0.007
**101165**	0.447	8.129	0.493	0.215	0.412	0.353	0.277	7.131	1.428	2.478	0.108	0.337	0.187	0.069	0.129	1.017	0.172	0.010
**101171**	0.719	8.249	0.711	0.195	0.695	0.573	0.426	11.257	1.993	5.110	0.153	0.476	0.298	0.110	0.236	0.684	0.404	0.022
**101214**	0.848	10.138	0.659	0.571	0.629	0.444	0.329	11.850	1.540	3.908	0.150	0.627	0.276	0.144	0.281	0.873	0.532	0.024
**101220**	0.911	8.608	0.600	0.899	0.601	0.388	0.339	11.168	1.581	3.277	0.177	0.565	0.249	0.120	0.267	0.835	0.468	0.027
**110111**	0.775	9.120	0.670	1.134	0.604	0.414	0.240	8.562	1.293	8.852	0.183	0.690	0.201	0.176	0.265	1.863	0.377	0.027
**110115**	0.792	7.389	0.505	0.963	0.450	0.349	0.305	8.127	1.402	1.957	0.149	0.508	0.215	0.123	0.196	1.669	0.280	0.041
**110125**	1.286	4.608	0.817	1.116	0.762	0.333	0.222	8.956	1.255	5.726	0.186	0.786	0.199	0.165	0.415	1.273	0.632	0.019
**110220**	1.279	9.606	0.855	2.230	0.847	0.457	0.340	10.960	1.533	4.609	0.185	0.570	0.273	0.111	0.254	0.734	0.419	0.050
**110319**	0.938	8.029	0.908	2.756	0.786	0.344	0.240	8.663	1.252	4.150	0.116	0.463	0.210	0.099	0.229	0.555	0.376	0.035

**110353**	0.579	6.728	0.957	1.290	0.873	0.501	0.386	8.875	1.805	1.852	0.104	0.384	0.258	0.091	0.137	0.859	0.207	0.037
**110354**	0.737	8.264	1.024	1.166	0.882	0.488	0.420	9.909	1.956	1.871	0.129	0.408	0.297	0.065	0.189	0.556	0.327	0.029
**110358**	0.880	7.711	0.632	1.171	0.581	0.445	0.350	9.189	1.725	1.616	0.143	0.535	0.289	0.118	0.265	0.816	0.455	0.038
**110361**	0.651	5.585	0.458	0.884	0.428	0.289	0.187	5.479	1.134	1.242	0.085	0.307	0.167	0.063	0.091	0.737	0.135	0.017
**110362**	0.714	6.064	0.480	1.660	0.472	0.369	0.247	7.304	1.325	1.363	0.083	0.369	0.208	0.090	0.166	0.642	0.298	0.026
**110955**	0.483	2.930	0.799	1.320	0.777	0.362	0.244	4.650	1.249	11.514	0.129	0.531	0.202	0.097	0.164	1.244	0.306	0.046
**111031**	0.950	8.075	0.583	1.122	0.550	0.366	0.241	9.933	1.393	4.142	0.14	0.645	0.245	0.098	0.282	0.873	0.564	0.017
**1112009**	0.528	5.136	0.896	1.548	0.786	0.287	0.230	7.697	1.230	8.398	0.084	0.301	0.259	0.059	0.155	0.326	0.315	0.017
**1203061**	0.644	6.468	0.778	1.041	0.637	0.276	0.239	9.655	1.208	10.424	0.062	0.338	0.288	0.023	0.166	0.513	0.272	0.027

Notes: 1: sweroside; 2: magnoflorine; 3: psoralen; 4: timosaponin BII; 5: isopsoralen; 6: epimedin A; 7: epimedin B; 8: epimedin C; 9: icariin; 10: asperosaponin VI; 11: isobavachin; 12: neobavaisoflavone; 13: icariside II; 14: psoralidin; 15: isobavachalcone; 16: bavachinin; 17: corylifol A; 18: tanshinone IIA.

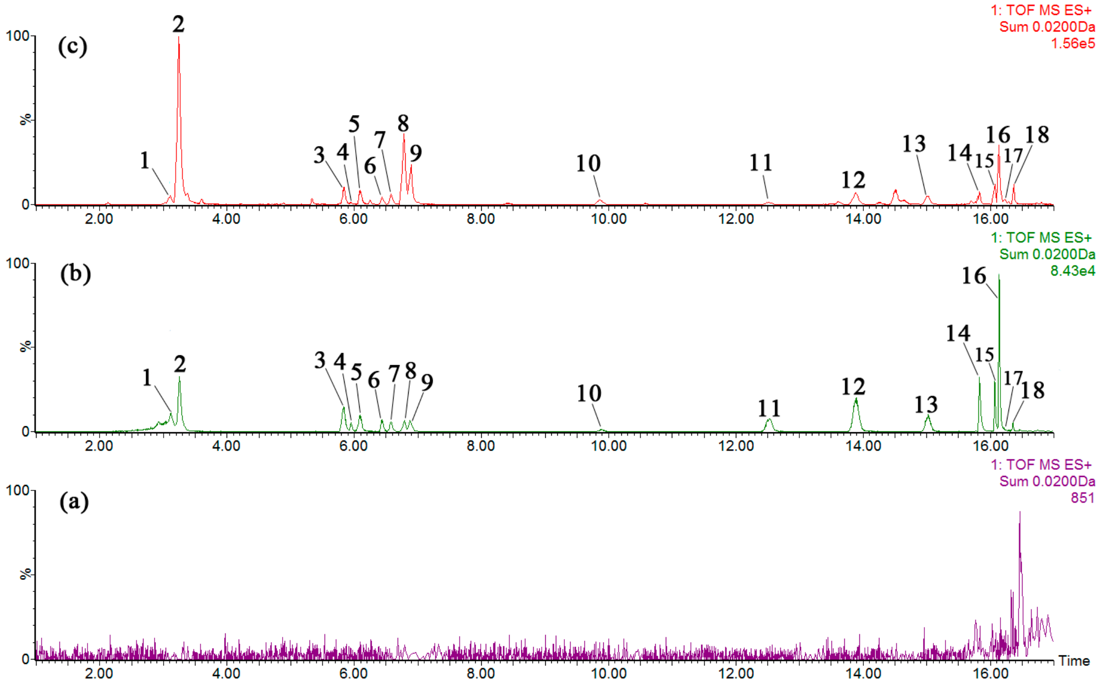

Figure S1 Extracted ion chromatograms (EICs) of 18 quantitative chemical markers

(a: Control solvent; b: Reference standards of 18 quantitative chemical markers; c: XLGB; 1: sweroside; 2: magnoflorine; 3: psoralen; 4: timosaponin BII; 5: isopsoralen; 6: epimedin A; 7: epimedin B; 8: epimedin C; 9: icariin; 10: asperosaponin VI; 11: isobavachin; 12: neobavaisoflavone; 13: icariside II; 14: psoralidin; 15: isobavachalcone; 16: bavachinin; 17: corylifol A; 18: tanshinone IIA)
